# Developing sport expertise in youth sport: a decision training program in basketball

**DOI:** 10.7717/peerj.7392

**Published:** 2019-08-13

**Authors:** Alexander Gil-Arias, Luis Garcia-Gonzalez, Fernando Del Villar Alvarez, Damián Iglesias Gallego

**Affiliations:** 1 Centre for Sport Studies, Rey Juan Carlos University, Alcorcón, Madrid, Spain; 2 Faculty of Health and Sport Sciences, University of Zaragoza, Huesca, Aragón, Spain; 3 Teacher Training College, University of Extremadura, Cáceres, Extremadura, Spain

**Keywords:** Cognitive expertise, Questioning, Video-feedback, Performance, Decision-making

## Abstract

**Background:**

This study has analyzed the impact of applying a decision training program, in which video-feedback and questioning were used, on the development of decision-making, skill execution and procedural knowledge in basketball players.

**Methods:**

Participants were eleven male players aged between 12 and 13 years old (*M*_age_ = 12.75, *SD*_age_ = .65), who were assigned to an experimental or control group within a pre-test/intervention test/retention test quasi-experimental design. The decision training program was applied over 11 weeks. Throughout this intervention, players had to analyze the causes and reasons for the decision made, using video feedback and questioning to this end. Decision-making and skill execution variables were analyzed using the French & Thomas (1987) observation instrument, while a validated questionnaire was used to assess procedural knowledge in basketball.

**Results:**

The results reported that sport expertise improved in players from the experimental group, who had significantly higher intervention test scores for successful decisions and skill executions when compared to players in the control group. In the intra-group analysis, the experimental group significantly improved in the intervention test compared to the pre-test, in terms of some of the variables of decision-making, skill execution and procedural knowledge.

**Discussion:**

These results reinforce the idea of including cognitive tools in training, such as video-feedback and questioning, to improve sport expertise in players’ formative stages, and presumably to improve their performance whilst maintaining decision training throughout time.

## Introduction

There is currently growing interest in acquiring accurate knowledge of the keys that determine sport expertise (Ibáñez Gijón et al., 2017). Different researchers have highlighted the importance of cognitive factors (e.g., decision-making and procedural knowledge), and skill execution components in sport expertise (Lex et al., 2015).

Grounded in the cognitive psychology approach, the sport expertise level largely depends on internal mental representations, and on the cognitive processes that mediate between the interpretation of a stimulus and the selection of a response (García-González et al., 2011). In this regard, cognitive processes play an important role in the development of open skills (e.g., pass and shoot in basketball), from the point of view of what to do at each moment of play. During a match, players must analyze the structural elements of the game action that determine sporting success. These elements include factors such as the scoreboard, opponents, location of teammates, offensive or defensive game patterns, etc. (Alves et al., 2013). This information is continuously stored and updated in the player’s memory, configuring its tactical knowledge base, thus enabling players to interpret the game situation and make successful decisions during the game (McPherson & Kernodle, 2007).

Analyzing decision-making in open sports (e.g., basketball) is especially relevant because this type of sport features a high degree of variability (Moran, 2012). In this regard, players must quickly understand what stimuli have a clear influence on decision-making, in order to select the most appropriate response in agreement with existing circumstances of the game (Johnson, 2006; Macquet, 2009).

Given the importance of cognitive expertise in sport (Lorains, Ball & MacMahon, 2013), and its relationship with performance level and skill execution, it is suggested that there is a need to develop training programs to improve cognitive and execution factors, designing representative tasks that include essential aspects of the game context (Marasso et al., 2014). Vickers (2007) developed a Decision Training Model (DTM) that was characterized by focusing the athletes’ training on establishing clearly tactical oriented tasks, to consider players’ behavioral interactions with the different environmental elements during the sporting action. This DTM posed the use of different tools, noteworthy among which are video-feedback and questioning. Through video-feedback, athletes can observe their decision-making in a training and competition situation and, therefore, they can identify the opposite team’s weaknesses and strengths, as well as improve the recognition of contextual factors (Groom, Cushion & Nelson, 2011; Nelson, Potrac & Groom, 2011). In this regard, video-feedback seems to be a useful tool to develop cognitive expertise, but it must be used with the help of a mentor or expert, because events occur quickly in the sporting scenario, making it difficult to interpret all the information. There is also little evidence to show that simply watching the video is effective in itself (Boyer et al., 2009). This allows directing the players’ attention toward specific and relevant information from the sports context (Potdevin et al., 2018; Vickers, 2007).

On the other hand, the main goal of questioning is to ask players questions with a high tactical component. This will enable them to explore new forms of interaction with the environment, and thus be able to effectively and accurately execute a technical-tactical skill (Harvey, Cope & Jones, 2016). Questioning helps guide players towards self-reflection, self-regulation and problem-solving (Chambers & Vickers, 2006; Oslin & Mitchell, 2006). In this case, when a mentor directs the player’s attention towards specific signs through questioning, results are significantly positive (Vickers, 2007). In this sense, the joint use of video-feedback and questioning appears to be an effective training strategy to improve sport expertise, especially at lower performance levels, where this could be more effective (Hodges, Chua & Franks, 2003; Raab, Masters & Maxwell, 2005).

Based on the aforementioned reasons, the decision training program presented in this study will combine video-feedback and questioning to improve players’ ability to select a correct response by analyzing their own actions of play, and to indirectly optimize their skill execution. Combining these two tools generates an increase in cognitive effort and makes it easier for athletes to understand the tactical and technical requirements of their sport (Vickers et al., 2004). In previous research, a combination of both tools has been applied to verify efficacy on decision-making (Gil-Arias et al., 2016; Moreno et al., 2011), skill execution (García-González et al., 2014), and tactical knowledge (Gil-Arias et al., 2015; Moreno et al., 2016). Research carried out to date has only focused on analyzing, from an independent perspective, the effect of applying a decision training program on some of the variables that determine sport expertise. Consequently, the current study aims to extend the above work through investigating the impact of applying a decision training program, in which video-feedback and questioning were used, on the development of decision-making, skill execution and procedural knowledge in basketball players. Derived from this purpose, the main hypothesis suggests that players participating in the decision training program would show greater improvement in successful decisions, skill execution and procedural knowledge than the control group.

## Materials & Methods

### Participants

Participants were twelve male basketball players aged between 12 and 13 years old (*M* = 12.75; *SD* = .65). One player, who initially took part in the current research, was removed because of an injury sustained during the study. All participants were exposed to the same formative process, which enabled us to control the “effect of training” variable, given that all the players belonged to the same club and to the same training group. They also all had the same coach and all trained five hours per week.

Participants were assigned to an experimental group (five players; age, *M* = 12.40, *SD* = .54; years of practice in basketball, *M* = 5.20, *SD* = .85) or a control group (six players; age, *M* = 12.66, *SD* = .51; years of practice in basketball, *M* = 5.32, *SD* = .92). Players were randomly assigned to the experimental or the control group, prior to applying the decision training program, ensuring that both groups were homogeneous in terms of study variables. To this end, Levene’s test for homogeneity of variances was performed, which showed that, before applying the decision training program, both groups were homogeneous in the decision-making (Levene’s statistic = 2.210; *p* = .158), skill execution (Levene’s statistic = 1.112; *p* = .253) and procedural knowledge (Levene’s statistic = 1.675; *p* = .322).

The research has been developed under the recommendations of the Helsinki Declaration. Informed written consent was obtained from all participants and their parents/guardians who were fully informed about the study. The research was authorized by the Institutional Review Board of the University of Extremadura (no: 63/2018).

### Variables

The independent variable was the decision training program based on the use of video-feedback and questioning. The intervention in the experimental group lasted for 11 weeks. The main aim of the decision training program was to improve the level of sport expertise (i.e., decision-making, skill execution and procedural knowledge), using video-feedback and questioning. The training program was designed to improve players’ ability to select a correct response by analyzing their own actions of play. Visualizing the situations selected allowed participants to observe their own decisions and, by means of open-ended questions (questioning), participants evaluated the suitability of the responses given. Players analyzed the consequences of their decisions and possible alternatives to them. The decision training program was applied by the last author, who had to guide the analysis of game situations by asking different questions with a high tactical component. The involvement of the researcher taking part in the sessions was of a subsidiary nature, guiding the analysis of the game situations through open questioning, attempting to make participants analyze their own decisions by themselves, evaluating the consequences, and designing alternatives to each action. In this regard, the researcher did not directly intervene in the analysis carried out by the player or give answers to the proposed questions.

The coach was not informed about the tactical aspects analyzed in each session. The intervention program was carried out in addition to the usual training of players from the experimental group. The main task was to complete an analysis of the decisions taken during a match, viewing successful and unsuccessful decisions in different real game actions.

**Figure 1:**
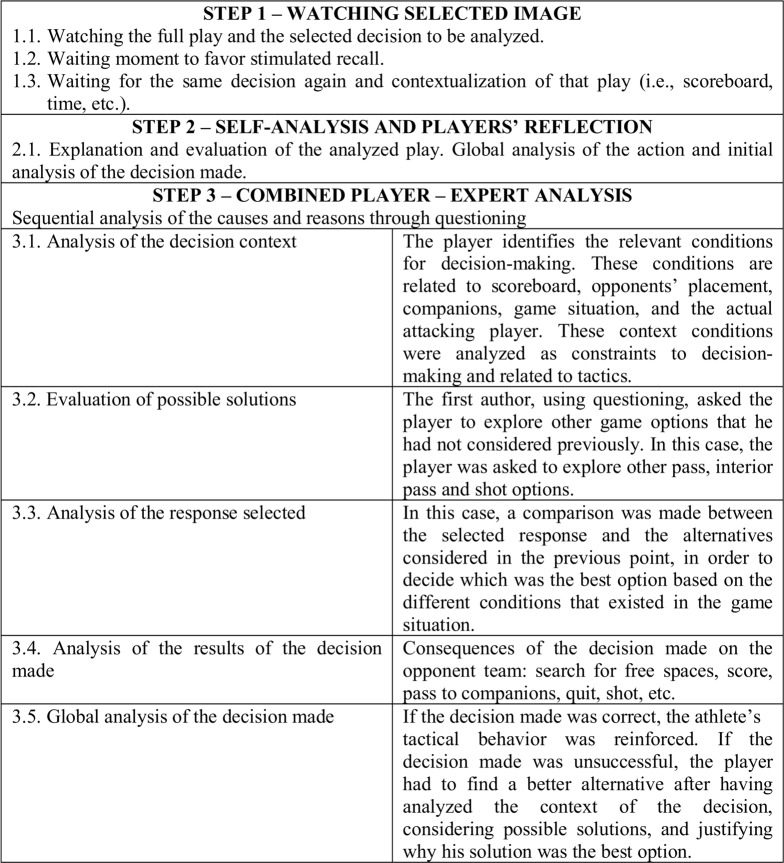
Sequence for the analysis of every decision made.

The features of the decision training program were as follows: (1) three successful decisions and three unsuccessful decisions were shown in each session to participants from the experimental group. Players had to learn the keys to make a successful decision and to be able to compare it to an unsuccessful decision. The order of these decisions was random in every session, to prevent players from knowing if the decision to be analyzed was successful or unsuccessful; (2) analysis of every selected decision, by means of video-feedback, showing the development of the whole play. The entire set of 6 actions to be analyzed comprised one complete session. The intervention structure was similar to previous studies that had been carried out on team sports (Moreno et al., 2016). The supervisor pointed out which decision was being analyzed within the whole play. This took about 45 min and was carried out according to the structure shown in Fig. 1.

The dependent variables were decision-making, skill execution and procedural knowledge. It is necessary to highlight that the decision-making and skill execution variables were related to technical pass and shot actions. The decision-making variable is defined as the process whereby players select one option (e.g., type of pass and shoot), to execute it at a specific moment and in a real game situation (Bar-Eli, Plessner & Raab, 2011). Skill execution is defined as the performance, outcome, or consequence provoked by the selected pass or shoot (Nielsen & McPherson, 2001). Procedural knowledge refers to the knowledge of how to do something. It is identified with “know how” and defined as a motor procedure (skill execution), and as a response selection procedure (which movement must be performed in a particular game situation) (McPherson, 2008).

### Instruments

The decision-making and skill execution assessment was based on external and indirect systematic observation. This methodology has been used in previous research to measure players’ decision-making and skill execution in real game situations (Van Maarseveen, Savelsbergh & Oudejans, 2018; Nielsen & McPherson, 2001). The observation was based on a real competition situation, which represents the influence of the environment on decision-making and skill execution (Raab & Köppen, 2009; Travassos et al., 2013). The French & Thomas (1987) observation instrument was used to assess the decision-making and skill execution of basketball players. Through this instrument, decision-making was coded as 1, if successful (e.g., *shot;* Any shot taken within a 6.25-meter radius of the basket when the player was open, a defensive player did not have an advantage; *pass;* A pass to a teammate who is open), or 0 if unsuccessful (e.g., *shot;* A shot taken off balance, balance not lost due to physical contact; *pass;* A pass made to a player who is closely guarded, defensive player positioned in the passing lane). Likewise, technical execution was coded as 1, if successful (e.g., *shot;* Successful shot; *pass;* Successful pass to a teammate), or 0 if unsuccessful (e.g., *shot;* Unsuccessful shot; *pass;* Pass that was too high, too far behind or in front of a teammate, out of bounds or at a teammate’s feet).

Decision-making was assessed by means of the percentage of successful decisions referring to the basketball player’s capacity to take appropriate decisions under specific conditions. Likewise, skill execution was assessed by means of the percentage of successful actions achieved in every match. To calculate the percentage of successful decisions and executions, the total number of decisions and executions were divided by the sum of the successful and unsuccessful decisions, and executions, and multiplied by 100 (Mitchell, Oslin & Griffin, 2006).

The observation process was carried out by one single outside observer, who holds a Sports Sciences degree and used to be a basketball coach with at least six years’ experience. The observer had to undergo a training process with an expert researcher, establishing a total of five 45-minute sessions in order to obtain suitable reliability in the decision-making and skill execution analysis. A sample of five matches was observed, that is, more than 10% the total sample (Tabachnick & Fidell, 2007). Reliability was calculated using Cohen’s Kappa statistic, reaching values of more than .80 in the five training sessions. Temporal reliability was also ensured, analyzing the same matches 10 days later, obtaining intra-observer reliability results of .86 for both variables.

A questionnaire was used to assess procedural knowledge in basketball. This instrument was adapted from the original by McGee & Farrow (1987). Validation resulted in a final version comprising 16 questions (Del Villar et al., 2004), with a suitable level of validity and reliability. Internal consistency values of above .70 were obtained by Cronbach’s alpha (Nunnally, 1978). Every question had one single correct answer out of a multiple choice of four options. The measurement of the procedural knowledge was calculated by the percentage of correct answers achieved by each participant in the questionnaire. This same instrument has been used in other research studies to evaluate cognitive expertise at difference expertise levels (Iglesias et al., 2005). The questionnaires were completed by the players in a closed room after a training session.

### Procedure

**Figure 2:**
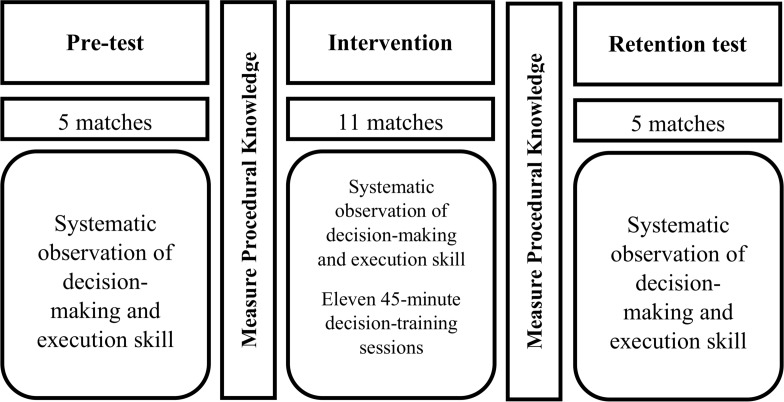
Timeline of the study. .

This study was conducted with 2 groups (i.e., experimental and control) at 3 times (i.e., pre-test, intervention test, and retention test). The study flow can be seen in Fig. 2. Each player played 16 basketball matches, which were divided into three experimental phases:

Phase A (pre-test): This phase lasted for five weeks. Decision-making and skill execution were assessed across five competition matches. Procedural knowledge was measured at the end of this phase. These initial measurements were tested for homogeneity and lack of differences between groups.

Phase B (intervention test): This second phase lasted for eleven weeks during which the decision training program was applied with eleven video-feedback and questioning sessions for every player belonging to the experimental group. These sessions were carried out after every match. Matches were played on Saturdays or Sundays. Each intervention session was held during the following 24 h, before athletes carried out their first weekly training. Players from the experimental group spent about 45 min on the decision training program in each session, while players from the control group spent the same time viewing decisions with the same characteristics. The control group also viewed six decisions (three successful decisions and three unsuccessful decisions) but they did not receive tactical information.

Phase C (retention test): This last phase lasted for five matches, when the decision training program was no longer applied, and whose purpose was to evaluate the degree of retention, in terms of decision-making and skill execution, in participants from the experimental group.

During the development of the study, all participants trained for the same time and competed in the same number of matches. The only difference was that players from the experimental group received the decision training program. Parallel to the application of the intervention, the decision-making and skill execution variables of players from both groups were assessed during the competition matches that took place at weekends. Procedural knowledge was measured once again at the end of the intervention phase. The measurement of procedural knowledge could not be carried out in the retention test phase, because the participants in the research were not available at that time.

### Statistical analysis

Version 24.0 of the statistical package for social sciences (SPSS, Armonk, NY, USA) was used for the data analysis. Results of the Shapiro–Wilks test (for samples of less than 50 individuals) revealed that data were not normally distributed (*p* < .05), which led to the use of non-parametric statistics. Mean and standard deviations were calculated for each group at each of the three different time points. The non-parametric Mann–Whitney *U* test was used in the inter-group analysis at the different moments of the research. The effect size was calculated separately for each variable by $r=Z/\sqrt{}N$ (Rosenthal & Dimatteo, 2001). The Wilcoxon signed-rank test was used to contrast the differences between the different moments of the research for each group (i.e., experimental group and control group). The level of statistical significance was established at *p* ≤ .05 (95% confidence interval).

## Results

### Descriptive statistics and inter-group analysis

No differences were found between the two groups in either the decision-making or the skill execution sub-variables before the decision training program was applied (see Table 1).

**Table 1:** Means, standard deviation and inter-group analysis for decision-making and skill execution variables in pre-test.

Variables	Experimental group		Control group		*U*	*Z*	*p*	*r*
	M	SD	M	SD				
General DM	76.80	3.03	76.92	8.25	13.00	−0.37	.715	0.10
Total shot DM	72.77	6.60	73.81	8.78	14.00	−0.18	.855	0.05
Interior shot DM	66.05	9.08	72.28	7.10	9.00	−1.09	.273	0.31
Outer shot DM	93.83	5.82	95.14	7.65	13.50	−0.30	.792	0.08
Total pass DM	80.83	2.37	80.03	14.81	12.00	−0.55	.584	0.15
Pass to zone DM	67.34	11.90	62.72	24.64	13.00	−0.37	.714	0.10
6.25 m pass DM	78.25	8.70	82.89	7.75	6.00	−1.65	.100	0.47
Far pass DM	87.23	7.13	91.97	5.83	9.00	−1.10	.273	0.31
General SE	64.17	8.09	65.19	9.50	15.00	0.00	1.00	0.00
Total shot SE	40.92	14.70	46.45	14.72	10.00	−0.91	.361	0.26
Interior shot SE	45.49	14.13	49.93	10.41	15.00	0.00	1.00	0.00
Outer shot SE	23.80	16.44	18.33	28.58	9.50	−1.03	.303	0.29
Total pass SE	87.41	2.00	83.92	9.42	11.00	−0.73	.465	0.21
Pass to zone SE	76.03	14.22	72.50	20.29	13.00	−0.37	.715	0.10
6.25 m pass SE	84.82	8.59	87.65	8.57	12.00	−0.55	.584	0.15
Far pass SE	92.94	7.50	93.22	5.50	13.00	−0.37	.712	0.10

**Notes.**

DM decision making SE skill execution

After the intervention, results showed that the experimental group presented a significantly higher mean percentage of successful decisions and execution than the control group in the following variables: general decision-making, decision-making in total shots, decision-making in interior shots, general skill execution, skill execution in total shots and skill execution in interior shots (see Table 2).

**Table 2:** Means, standard deviation and inter-group analysis for decision-making and skill execution variables in intervention test.

Variables	Experimental group		Control group		*U*	*Z*	*p*	*r*
	M	SD	M	SD				
General DM	87.90	1.79	73.30	5.66	0.00	−2.74	**.006**	0.79
Total shot DM	84.68	2.38	62.26	9.00	0.00	−2.74	**.006**	0.79
Interior shot DM	82.41	4.89	59.68	8.08	0.00	−2.74	**.006**	0.79
Outer shot DM	89.90	5.90	93.33	14.91	7.00	−1.23	.219	0.35
Total pass DM	91.11	2.22	84.32	6.98	6.50	−1.56	.120	0.45
Pass to zone DM	83.95	4.96	72.70	11.31	6.00	−1.64	.100	0.47
6.25 m pass DM	93.09	4.35	86.23	15.22	13.50	−0.27	.784	0.07
Far pass DM	93.74	4.95	91.89	4.66	11.00	−0.73	.465	0.21
General SE	75.14	3.34	67.40	3.55	1.00	−2.56	**.011**	0.73
Total shot SE	57.44	6.57	48.19	5.76	4.00	−2.01	**.045**	0.58
Interior shot SE	61.17	7.27	50.50	8.08	4.00	−2.01	**.045**	0.58
Outer shot SE	41.95	11.86	27.50	29.11	9.00	−0.73	.465	0.21
Total pass SE	92.83	1.72	86.61	4.79	5.50	−1.74	.082	0.50
Pass to zone SE	87.56	8.87	76.18	13.03	9.00	−1.10	.273	0.31
6.25 m pass SE	94.25	3.17	89.22	10.58	12.00	−0.55	.584	0.15
Far pass SE	93.27	4.45	92.13	4.30	12.00	−0.55	.584	0.15

**Notes.**

DM decision making SE skill execution

No significant differences were obtained between the two groups in any of the decision-making and skill execution sub-variables in the retention test (see Table 3).

**Table 3:** Means, standard deviation and inter-group analysis for decision-making and skill execution variables in retention test.

Variables	Experimental group		Control group		*U*	*Z*	*p*	*r*
	M	SD	M	SD				
General DM	90.67	6.11	78.06	12.09	6.00	−1.64	.100	0.47
Total shot DM	90.92	9.72	70.49	19.89	6.50	−1.56	.120	0.45
Interior shot DM	86.31	13.20	64.49	23.28	6.50	−1.56	.120	0.45
Outer shot DM	97.67	3.25	91.67	13.95	14.00	−0.21	.833	0.06
Total pass DM	90.36	6.95	85.64	5.96	9.00	−1.10	.273	0.31
Pass to zone DM	79.47	20.40	66.60	28.31	11.50	−0.64	.521	0.18
6.25 m pass DM	89.20	10.02	82.95	7.44	9.00	−1.10	.273	0.31
Far pass DM	96.81	2.81	94.33	5.05	11.00	−0.74	.461	0.21
General SE	71.06	7.52	64.24	9.73	11.00	−0.73	.465	0.21
Total shot SE	52.49	14.52	41.72	20.09	11.00	−0.73	.465	0.21
Interior shot SE	57.98	16.37	50.16	16.42	9.00	−1.10	.272	0.31
Outer shot SE	40.87	14.75	22.22	39.20	5.50	−1.76	.079	0.50
Total pass SE	89.62	5.81	86.75	4.45	8.00	−1.28	.201	0.36
Pass to zone SE	76.97	18.92	67.01	23.97	13.00	−0.37	.715	0.10
6.25 m pass SE	87.49	8.08	86.51	8.67	14.00	−0.18	.855	0.05
Far pass SE	96.83	2.37	94.33	5.05	10.00	−0.92	.357	0.26

**Notes.**

DM decision making SE skill execution

In procedural knowledge, no significant differences were obtained between the control group and the experimental group in the pre-test or in the intervention test (see Table 4).

**Table 4:** Means, standard deviation and inter-group analysis for procedural knowledge variable.

Variables	Experimental group		Control group		*U*	*Z*	*p*	*r*
	M	SD	M	SD				
Pre-test PK	80.00	6.85	86.46	7.31	7.50	−1.44	.150	0.41
Intervention test PK	93.75	6.25	88.54	6.14	8.50	−1.10	.273	0.31

**Notes.**

PK procedural knowledge

### Descriptive statistics and intra-group analysis

A significant increase was found in the experimental group from pre-test to intervention test in the following variables: general decision-making, decision-making in total shots, decision-making in interior shots, decision-making in total pass, decision-making in pass to zone, general skill execution, skill execution in total shots, skill execution in interior shots and skill execution in total passes. What is more, a significant increase was found from intervention test to retention test in decision-making in far passes. In contrast, a significant decrease was found from intervention test to retention test in the following variables: total pass decision-making and 6.25 m pass skill execution. A significant increase was also found from pre-test to retention test in general decision-making, total shot decision-making and interior shot decision-making. No significant differences were found in interior shot decision-making, 6.25 m pass decision-making, outer shot skill execution, pass-to-zone skill execution and far pass skill execution (see Table 5).

**Table 5:** Means, standard deviation and pairwise comparison for decision-making and skill execution variables—Experimental group.

Variables experimental group	Pre-test		Intervention test		Retention test		*Dif.*
	M	SD	M	SD	M	SD	
General DM	76.80	3.03	87.90	1.79	90.67	6.11	**a,c**
Total shot DM	72.77	6.60	84.68	2.38	90.92	9.72	**a, c**
Interior shot DM	66.05	9.08	82.41	4.89	86.31	13.20	**a, c**
Outer shot DM	93.83	5.82	89.90	5.90	97.67	3.25	nd
Total pass DM	80.83	2.37	91.11	2.22	90.36	6.95	**a,b**
Pass to zone DM	67.34	11.90	83.95	4.96	79.47	20.40	**a**
6.25 m pass DM	78.25	8.70	93.09	4.35	89.20	10.02	nd
Far pass DM	87.23	7.13	93.74	4.95	96.81	2.81	b
General SE	64.17	8.09	75.14	3.34	71.06	7.52	**a**
Total shot SE	40.92	14.70	57.44	6.57	52.49	14.52	**a**
Interior shot SE	45.49	14.13	61.17	7.27	57.98	16.37	**a**
Outer shot SE	23.80	16.44	41.95	11.86	40.87	14.75	nd
Total pass SE	87.41	2.00	92.83	1.72	89.62	5.81	**a**
Pass to zone SE	76.03	14.22	87.56	8.87	76.97	18.92	nd
6.25 m pass SE	84.82	8.59	94.25	3.17	87.49	8.08	b
Far pass SE	92.94	7.50	93.27	4.45	96.83	2.37	nd

**Notes.**

DM decision making SE skill execution Dif. differences a differences between pre-test and intervention test b differences between pos *t*-test and retention test c differences between pre-test and retention test nd no differences between any phase or test time

Note: all differences expressed are *p* < .05.

A significant decrease was found in the control group from pre-test to intervention test in the following variables: total shot decision-making and interior shot decision-making (see Table 6).

**Table 6:** Means, standard deviation and pairwise comparison for decision-making and skill execution variables—Control Group.

Variables control group	Pre-test		Intervention test		Retention test		*Dif.*
	M	SD	M	SD	M	SD	
General DM	76.92	8.25	73.30	5.66	78.06	12.09	nd
Total shot DM	73.81	8.78	62.26	9.00	70.49	19.89	**a**
Interior shot DM	72.28	7.10	59.68	8.08	64.49	23.28	**a**
Outer shot DM	95.14	7.65	93.33	14.91	91.67	13.95	nd
Total pass DM	80.03	14.81	84.32	6.98	85.64	5.96	nd
Pass to zone DM	62.72	24.64	72.70	11.31	66.60	28.31	nd
6.25 m pass DM	82.89	7.75	86.23	15.22	82.95	7.44	nd
Far pass DM	91.97	5.83	91.89	4.66	94.33	5.05	nd
General SE	65.19	9.50	67.40	3.55	64.24	9.73	nd
Total shot SE	46.45	14.72	48.19	5.76	41.72	20.09	nd
Interior shot SE	49.93	10.41	50.50	8.08	50.16	16.42	nd
Outer shot SE	18.33	28.58	27.50	29.11	22.22	39.20	nd
Total pass SE	83.92	9.42	86.61	4.79	86.75	4.45	nd
Pass to zone SE	72.50	20.29	76.18	13.03	67.01	23.97	nd
6.25 m pass SE	87.65	8.57	89.22	10.58	86.51	8.67	nd
Far pass SE	93.22	5.50	92.13	4.30	94.33	5.05	nd

**Notes.**

DM decision making SE skill execution Dif. differences a differences between pre-test and intervention test b differences between pos *t*-test and retention test c differences between pre-test and retention test nd no differences between any phase or test time

Note: all differences expressed are *p* < .05.

Regarding procedural knowledge, a significant increase was found in the experimental group from pre-test to intervention test. In contrast, no significant differences were found in the control group (see Table 7).

**Table 7:** Means, standard deviation and intra-group analysis for procedural knowledge variable.

Variables	Pre-test		Intervention test		Wilcoxon test (Z)	*p*
	M	SD	M	SD		
Experimental Group - PK	80.00	6.85	93.75	6.25	−2.06	**.039**
Control Group - PK	86.46	7.31	88.54	6.14	−0.54	.589

**Notes.**

PK procedural knowledge

## Discussion

In scientific literature, different researchers have determined that in open sports, like basketball, expertise is comprised of a cognitive component and a technical execution component (Faubert, 2013; Roca, Ford & Memmert, 2018). Most studies on this research topic have only focused on analyzing, from an independent perspective, the effect of applying a decision training program on some of the variables that determine sport expertise, but no research has been found where a decision training program has been applied to verify its effect on the different components that determine sport expertise. Consequently, our interest was to examine the effect of applying a decision training program, in which video-feedback and questioning were used, on the development of decision-making, skill execution and procedural knowledge in basketball players. Derived from this purpose, it was hypothesized that players participating in the decision training program would show greater improvement in successful decisions, skill execution and procedural knowledge than the control group.

In this study, the results have proven the efficacy of the decision training program based on the use of video-feedback and questioning, given that, after applying the intervention program, the experimental group showed a significantly higher number of successful decisions than the control group. In this regard, the decision training program designed for this research, and based on video-feedback and questioning, had a significant effect on players’ decision making (Hodges, Chua & Franks, 2003; Vickers, 2007). The effectiveness of the decision training program has been observed in previous research developed with young athletes, both in tennis (García-González et al., 2013) and in volleyball (Gil-Arias et al., 2016). These findings are due to the characteristics of the decision training program, which allowed us to analyze the different tactical aspects of the analyzed game situation, in addition to making an in-depth analysis of the most effective decisions (Vickers, 2007). Thus, the application of the decision training program meant that athletes had to focus their attention on relevant aspects of the game situation (e.g., location of teammates, position of players in the opposite team) to carry out a more sophisticated interpretation of the tendencies, strengths and weaknesses of the opposite team (Mann et al., 2007; Murphy, Jackson & Williams, 2018). This implies selecting the most suitable actions (e.g., pass and shot) to make it difficult for opponents to play their own game.

In relation to skill execution, the results determined that participants in the experimental group showed a significantly higher number of successful skill executions than control group players in the intervention test. In this sense, the decision training program focused on analyzing the decision made, not only in terms of the players’ tactical behavior, but also in terms of other factors that influence sport expertise, such as technical execution (Phillips et al., 2010). In this regard, there are sundry research studies that suggest a relationship between decision-making and technical execution, in such a way that the more successful decisions made, and effective executions carried out, the higher the performance (Hastie, Sinelnikov & Guarino, 2009). These research studies justify that the improvements obtained in participants from the experimental group, in relation to decision-making, may have an indirect effect on the quality of technical execution, since players who have greater capacity to satisfactorily solve tactical situations, are able to modify and adapt their motor execution more effectively, in agreement with the game context, in order to achieve higher performance (Nielsen & McPherson, 2001). Based on the results obtained, it is worth highlighting the effectiveness of the decision training program in improving sport expertise in young athletes. These findings have been stated in previous research studies that have been performed in other sports using these same tools (Chambers & Vickers, 2006; Moreno et al., 2008; Vickers, 2007). In this way, the decision training program applied in this research is not only effective regarding which decisions to make, but also regarding how to execute the decision made (Raab, Masters & Maxwell, 2005).

In relation to procedural knowledge, the study results have determined that, once the application of the intervention program was completed, participants from the experimental group showed a significantly higher score in procedural knowledge than the measure taken initially. In contrast, no significant changes were observed in the control group between the pre-test and the intervention test in terms of procedural knowledge. These results are consistent with previous research, in which procedural knowledge was measured by questionnaires after applying a decision training program (Blomqvist, Luhtanen & Laakso, 2001). This greater development of procedural knowledge allows athletes to make decisions that are more appropriate in real game situations, and to make adjustments in those situations that are temporarily conditioned to selecting an appropriate response (Araujo, Afonso & Mesquita, 2011; Loffing & Hagemann, 2014). Thus, players have greater capacity to solve game problems more effectively, given that they can access more sophisticated knowledge and use more specific strategies that allow them to decide and execute better (Roca et al., 2011).

According to the results obtained in this research, stimulating the capacity to analyze the decision made has proven to be an effective training tool to produce improvements in elements related to sport expertise. The effect produced by the decision training program in the different dependent variables is due to the characteristics of the intervention protocol, since it complies with some of the guidelines established by McPherson (2008) for the optimization of tactical-decisional aspects. Among these guidelines, we can highlight the following: frequent analysis of tactical performance in competition situations; helping to recognize contextual factors; reinforcing appropriate decisions and not just appropriate executions; providing opportunities for the analysis of tactical behavior, not only of oneself, but also of the opponent team; using new technologies for the development of action profiles based on the analysis of the opponent’s strengths and weaknesses. In this regard, the development and application of a decision training program, based on the use of video-feedback and questioning, favors the development of more critical thinking in athletes (Larkin et al., 2018; Sawiuk, Taylor & Groom, 2016), as there is an increase in time spent on tasks considered to be essential to achieve sport expertise (Catteeuw et al., 2009). Therefore, it may be important for coaches to include the use of cognitive tools, such as video-feedback and questioning, in their weekly training plans to make an audiovisual analysis of the decisions taken, thus permitting a faster increase of the different components of cognitive expertise, due to the summation character of cognitive experience and motor experience.

We can note several strengths of the current study. First, instruments with sound validity and reliability were used in the research to collect data. Second, all players belonged to the same training group, all athletes had the same coach, and they trained the same number of hours a week. They were also presented with the same cognitive content and training experiences. Therefore, we were able to control the training effect variable. Third, researchers used a quasi-experimental design, which allowed them to control homogeneity of both groups in the study variables, prior to the application of the decision training program. Thus, we can report that the results obtained in the current study are not due to the initial differences, and therefore we can conclude that any significant differences obtained in the current study can be attributed to the decision training program. Fourth, unlike other studies, in which the intervention program was applied to a certain game action, this research analyzed the effect of the decision training program on the different possibilities of both the pass and the shot.

Despite the aforementioned strengths, several limitations and future research directions should be considered. First, the use of a small sample and only one team limit the capacity to extrapolate the results. Therefore, future research can extend the current sample increasing the number of participants from different teams that have the same training conditions, thus providing greater power to detect significant differences. Second, in this study, procedural knowledge variable was measured by a previously validated questionnaire, which is characterized by a more dispositional measurement. Consequently, it would be valuable to replicate this research with verbal protocols, also used in other research (McPherson & Kernodle, 2007), which allows access to more situational and contextual aspects of the game actions.

## Conclusions

Among the findings from the current study, we would like to highlight the effect produced by the decision training program on sport expertise, and the need to use cognitive tools, such as video-feedback and questioning, in a training context, due to their importance during the athletes’ training process, and the strong relationship between cognitive and performance variables in sport. In this regard, it is very important to carry out good planning when these types of tools are included in training contexts, to generate a pathway in the player training process, where they can acquire not only technical development, but also cognitive and tactical development in the sport. In this sense, more intervention studies are necessary, since, although they involve greater research effort, they provide a great opportunity to afford scientific evidence to the training process.

##  Supplemental Information

10.7717/peerj.7392/supp-1 Supplemental Information 1 Questionnaire and Observation

## Additional Information and Declarations

### Data Availability

The following information was supplied regarding data availability: The Questionnaire and Observation data is available at Figshare: Gil-Arias, Alexander (2019): Developing sport expertise in youth sport: a decision training program in basketball. figshare. Dataset. https://doi.org/10.6084/m9.figshare.7732259.v1.
